# A de novo molecular generation method using latent vector based generative adversarial network

**DOI:** 10.1186/s13321-019-0397-9

**Published:** 2019-12-03

**Authors:** Oleksii Prykhodko, Simon Viet Johansson, Panagiotis-Christos Kotsias, Josep Arús-Pous, Esben Jannik Bjerrum, Ola Engkvist, Hongming Chen

**Affiliations:** 1 Hit Discovery, Discovery Sciences, Biopharmaceutical R&D, AstraZeneca, Gothenburg, Sweden; 2 Department of Chemistry and Biochemistry, University of Bern, Bern, Switzerland; 3 Department of Computer Science and Engineering, Chalmers University of Technology, Gothenburg, Sweden; 4 Chemistry and Chemical Biology Centre, Guangzhou Regenerative Medicine and Health-Guangdong Laboratory, Science Park, Guangzhou, China

**Keywords:** Molecular design, Autoencoder networks, Generative adversarial networks, Deep learning

## Abstract

Deep learning methods applied to drug discovery have been used to generate novel structures. In this study, we propose a new deep learning architecture, LatentGAN, which combines an autoencoder and a generative adversarial neural network for de novo molecular design. We applied the method in two scenarios: one to generate random drug-like compounds and another to generate target-biased compounds. Our results show that the method works well in both cases. Sampled compounds from the trained model can largely occupy the same chemical space as the training set and also generate a substantial fraction of novel compounds. Moreover, the drug-likeness score of compounds sampled from LatentGAN is also similar to that of the training set. Lastly, generated compounds differ from those obtained with a Recurrent Neural Network-based generative model approach, indicating that both methods can be used complementarily.
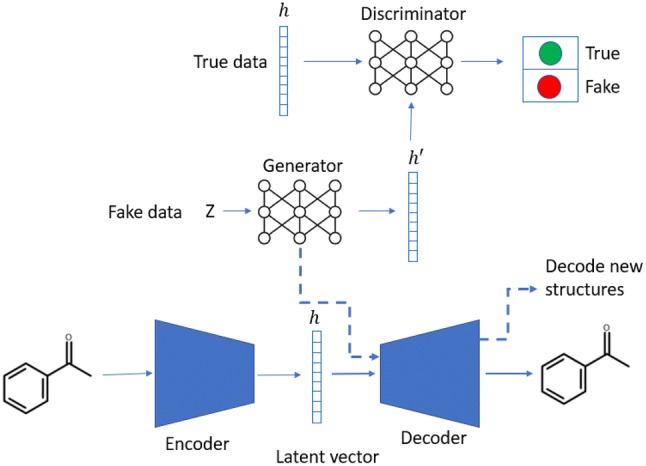

## Introduction

There has been a surge of deep learning methods applied to cheminformatics in the last few years [1–5]. Whereas much impact has been demonstrated in deep learning methods that replace traditional machine learning (ML) approaches (e.g., QSAR modelling [6]), a more profound impact is the application of generative models in de novo drug design [7–9]. Historically, de novo design was performed by searching virtual libraries based on known chemical reactions alongside a set of available chemical building blocks [10] or by using transformational rules based on the expertise of medicinal chemists to design analogues to a query structure [11]. While many successes using these techniques have been reported in literature [12], it is worthwhile to point out that these methods rely heavily on predefined rules of structure generation and do not have the concept of learning prior knowledge on how drug-like molecules should be. In contrast, deep generative models learn how to generate molecules by generalizing the probability of the generation process of a large set of chemical structures (i.e., training set). Then, structure generation is basically a sampling process following the learned probability distribution [7, 8, 13, 14]. It is a data-driven process and requires very few predefined rules.

Early attempted architectures were inspired by the deep learning methods used in natural language processing (NLP) [7, 15]. A recurrent neural network (RNN) trained with a set of molecules represented as SMILES strings [16] is able to generate a much bigger chemical space than the training set. Later on, the REINVENT method was proposed, which combines RNNs with reinforcement learning to generate structures with desirable properties [8]. Another architecture, the variational autoencoder (VAE), was also shown to generate novel chemical space [9, 17]. This architecture is comprised of an encoder, that converts the molecule to a latent vector representation and a decoder, from which the latent representation tries to generate the input molecule again. By changing the internal latent representation and decoding it, new chemical space can be obtained. More studies followed that improved the architecture, in both making it more robust and improving the quality of the latent representation generated [18–20]. One special mention is the use of randomized SMILES [14, 21, 22]. Instead of using a unique SMILES representation for each molecule, different representations are used in every stage of the training. With this improvement, the quality of the chemical space generated in both RNNs and VAEs is much higher and the models tend to overfit much less. Besides the SMILES string based de novo structure generation methods, methods of generating molecules based on molecular graphs have also been proposed and, by using them, molecules can be directly generated step-by-step as molecular graphs [23–26].

Generative adversarial neural (GAN) networks [27] have become a very popular architecture for generating highly realistic content [28]. A GAN has two components, a generator and a discriminator, that compete against each other during training. The generator generates artificial data and the discriminator attempts to distinguish it from real data. The model is trained until the discriminator is unable to distinguish the artificial data from the real data. The first use in molecule generation was ORGAN [29] and its improved version, ORGANIC [30]. The former was tested with both molecular generation as well as musical scores, whereas the latter was targeted directly at inverse design of molecules. ORGANIC had trouble optimizing towards the discrete values from the Lipinski Rule of Five [31] heuristic score but showed some success in optimizing the QED [11] score. An algorithm combining GAN with RL was also used in RANC [32] and ATNC [33] where the central RNN was substituted by a differential neural computer (DNC) [34], a more advanced recurrent neural network architecture. The authors demonstrated that DNC-based architectures can handle longer SMILES and generate more diversity.

In this study, a new molecular generation strategy is described which combines an autoencoder and a GAN. The difference between this method and previous GAN methods such as ORGANIC and RANC is that the generator and discriminator network do not use SMILES strings as input, but instead n-dimensional vectors derived from the code-layer of an autoencoder trained as a SMILES heteroencoder [35]. This allows the model to focus on optimizing the sampling and not worry about SMILES syntax issues. The decoder part of a pretrained heteroencoder [22] neural network was used to translate the generated n-dimensional vector into molecular structures. We first trained the GAN on a set of ChEMBL [36] compounds and, after training, the GAN model was able to generate drug-like structures. Next, additional GAN models were trained on three target specific datasets (corresponding to EGFR, HTR1A and S1PR1 targets). Our results show that these GAN model can generate compounds which are similar to the ones in the training set but are still novel structures. We envision the LatentGAN to be a useful tool for de novo molecule design.

## Methods and materials

### Heteroencoder architecture

**Fig. 1:**
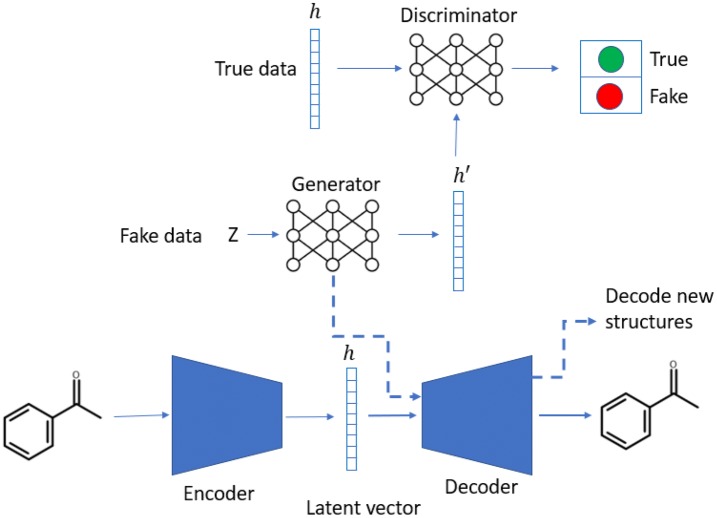
Workflow of the LatentGAN. The latent vectors generated from the encoder part of the heteroencoder is used as the input for the GAN. Once the training of the GAN is finished, new compounds are generated by first sampling the generator network of the GAN and then converting the sampled latent vector into a molecular structure using the decoder component of the heteroencoder

A heteroencoder is an autoencoder architecture trained on pairs of different representations of the same entity, i.e. different non-canonical SMILES of the same molecule. It consists of two neural networks, namely, the encoder and decoder, which are jointly trained as a transformation pipeline. The encoder is responsible for translating one-hot encoded SMILES strings into a numerical latent representation whereas the decoder accepts this latent representation and attempts to reconstruct one of the possible non-canonical SMILES string that it represents. The implementation followed the architecture previously reported in [22] with some changes (Fig. 1, bottom).

Initially, the one-hot encoded SMILES string is propagated through a two-layer bidirectional encoder with 512 Long Short-Term Memory [26] units per layer, half of which are used for the forward and half for the backward direction. The output of both directions is then concatenated and input to a feed-forward layer with 512 dimensions. As a regularizing step during training, the resulting vector is perturbed by applying additive zero-centered gaussian noise with a standard deviation of 0.1. The latent representation of the molecule is fed to a feed-forward layer, the output of which is copied and inserted as hidden and cell states to a four-layer unidirectional LSTM RNN decoder with the same specifications as the encoder. Finally, the output of the last layer is processed by a feed-forward layer with softmax activation, to return the probability of sampling each character of the known character set of the dataset. Batch normalization with a momentum value of 0.9 [37] is applied on the output of every hidden layer, except for the gaussian noise layer.

The heteroencoder network was trained for 100 epochs with a batch size of 128 and using a constant learning rate of 10^−3^ for the first 50 epochs and an exponential decay following that, reaching a value of 10^−6^ in the final epoch. The decoder was trained using the teacher’s forcing method [38]. The model was trained using the decoding loss function of categorial cross entropy between the decoded and the training SMILES. After training the heteroencoder, the noise layer is deactivated, resulting in a deterministic encoding and decoding of the GAN training and sampled sets.

### The GAN architecture

A Wasserstein GAN with gradient penalty (WGAN-GP) [39, 40] was chosen as a GAN model. Every GAN consists of two neural networks, generator and discriminator that train simultaneously (Fig. 1, top). First, the discriminator, usually called the critic in the context of WGANs, tries to distinguish between real data and fake data. It is formed by three feed-forward layers of 256 dimensions each with the leaky ReLU [41] activation function between, except for the last layer where no activation function was used. Second, the generator consists of five feed-forward layers of 256 dimensions each with batch normalization and leaky ReLU activation function between each.

### Workflow for training and sampling of the LatentGAN

The heteroencoder model was first pre-trained on the ChEMBL database for mapping structures to latent vectors. To train the full GAN model, first the latent vector *h* of the training set was generated using the encoder part of the heteroencoder. Then, it was used as the true data input for the discriminator, while a set of random vectors sampled from a uniform distribution were taken as fake data input to the generator. For every five batches of training for the discriminator, one batch was assigned to train the generator, so that the critic is kept ahead while providing the generator with higher gradients. Once the GAN training was finished, the Generator was sampled multiple times and the resulting latent vectors were fed into the decoder to obtain the SMILES strings of the underlying molecules.

### Dataset and machine learning models for scoring

The heteroencoder was trained on 1,347,173 SMILES from the ChEMBL [36] dataset. This is a subset of ChEMBL 25 without duplicates that has been standardized using the MolVS [42] v0.1.1 package with respect to the fragment, charge, isotope, stereochemistry and tautomeric states. The set is limited to SMILES of containing only [H, C, N, O, S, Cl, Br] atoms and a total of 50 heavy atoms or less. Furthermore, molecules known to be active towards DRD2 were removed as part of an experiment for the heteroencoder (the process of which can be found at [35], which uses the same decoder model, but not the encoder). A set of randomly selected 100,000 ChEMBL compounds were later selected for training a general GAN model. Moreover, three target datasets (corresponding to EGFR, S1PR1 and HTR1A) were extracted from ExCAPE-DB [43] for training target specific GANs. The ExCAPE-DB datasets were then clustered into training and test sets so that chemical series were assigned either to the training or to the test set (Table 1). To benchmark the performance of the targeted models, RNN based generative models for the three targets were also created by first training a prior RNN model on the same ChEMBL set used for training the heteroencoder model and then using transfer learning [7] on each focused target set. Target prediction models were calculated for each target using the Support vector machine learning (SVM) implementation in the Scikit-learn [44] package and the 2048-length FCFP6 fingerprint were calculated using RDKit [45].

**Table 1:** Targeted data set and the performance of the SVM models

Target	Training set	Test set	SVM model	
			ROC-AUC	Kappa value
EGFR	2949	2326	0.850	0.56
HTR1A	48,283	23,048	0.993	0.90
S1PR1	49,381	23,745	0.995	0.91

Training set size (training set), test set size (test set), receiver operating characteristic area under the curve (ROC-AUC), kappa value

### Related works

A related architecture to the LatentGAN is the Adversarial Autoencoder (AAE) [46]. The AAE uses a discriminator to introduce adversarial training to the autoencoder and is trained typically using a 3-step training scheme of (a) discriminator, (b) encoder, (c) encoder and decoder, compared to the LatentGANs 2-step training. The AAE have been used in generative modeling of molecules to sample molecular fingerprints using additional encoder training steps [47], as well as SMILES representations [48, 49]. In other application areas, Conditional AAEs with similar training schemes have been applied to manipulate images of faces [50]. For the later application, approaches that have utilized multiple discriminators have been used to combine conditional VAEs and conditional GANs to enforce constraints on the latent space [51] and thus increase the realism of the images.

## Results and discussion

### Training the heteroencoder

The heteroencoder was trained on the 1,347,173 ChEMBL dataset compounds for 100 epochs. SMILES generated validity for the whole training set was 99% and 18% of the molecules were not reconstructed properly. Notice that the reconstruction error corresponds to decoding to a valid SMILES that belongs to a different compound; reconstruction to a different SMILES of the same molecule is not counted as an error. Test set compounds were taken as input to the encoder and their latent values were calculated and then decoded to SMILES string, the validity and reconstruction error of test set are 98% and 20% respectively (Table 2).

**Table 2:** The performance of heteroencoder in both the training and test sets

Dataset	# compounds	Validity (%)	Reconstruction error (%)
Training set	974,105	99	18
Test set	10,823	98	20

Percent of valid SMILES strings generated by the decoder (validity), percent of molecules not reconstructed correctly from valid SMILES (reconstruction error)

### Training on the ChEMBL subset

**Fig. 2:**
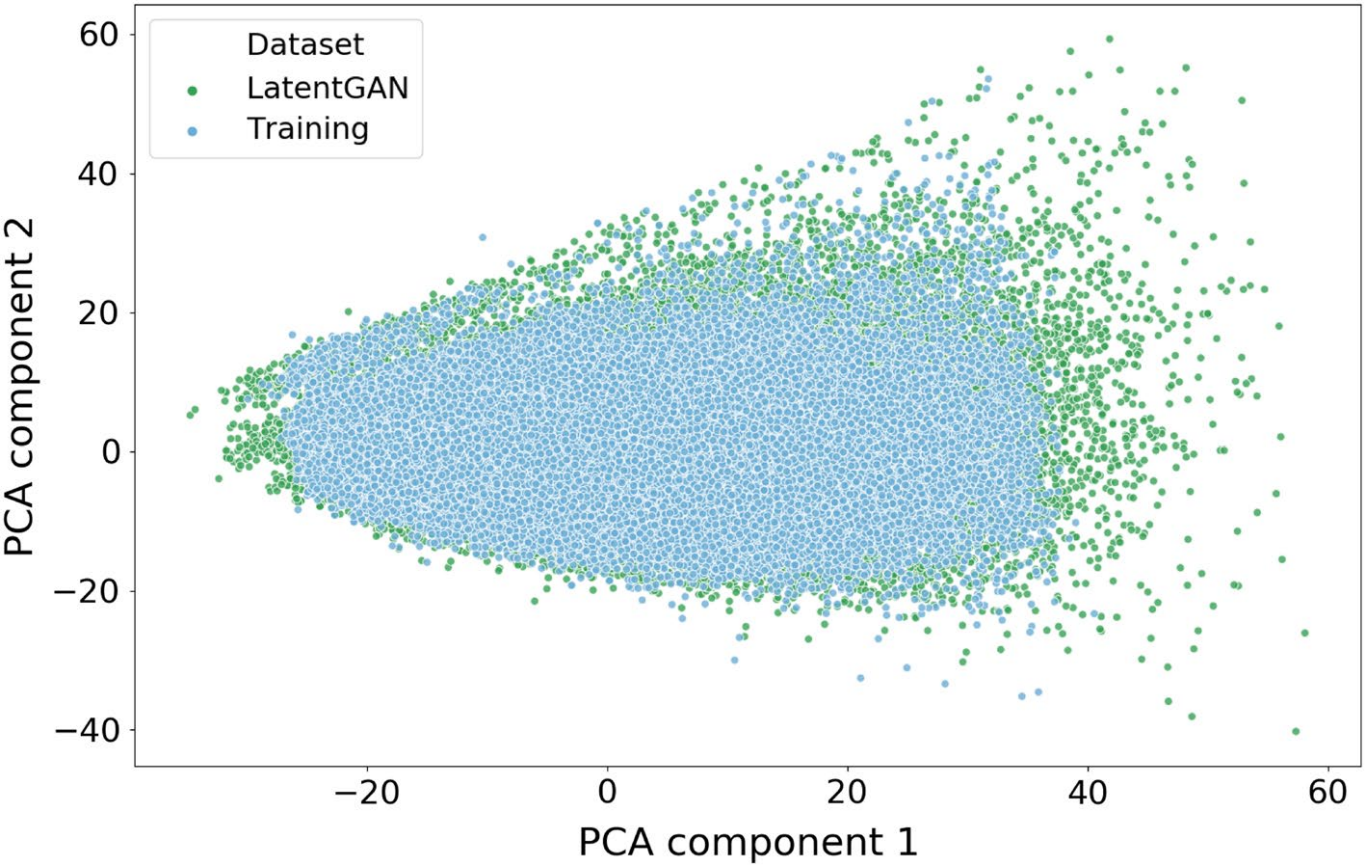
Plot of the first two PCA components (explained variance 74.1%) of a set of 200,000 generated molecules from the ChEMBL LatentGAN model using the MQN fingerprint

A LatentGAN was trained on a randomly selected 100,000 ChEMBL subset with the objective of obtaining drug-like compounds. The model was trained for 30,000 epochs until both discriminator and generator models had converged. Next, 200,000 compounds were generated from the LatentGAN model and were compared with the 100,000 ChEMBL training compounds to examine the coverage of the chemical space. The MQN [52] fingerprint was generated for all compounds in both sets and the top two principal components of a PCA were plotted (Fig. 2) and shows how both compound sets cover a similar chemical space.

### Training on the biased dataset

**Fig. 3:**
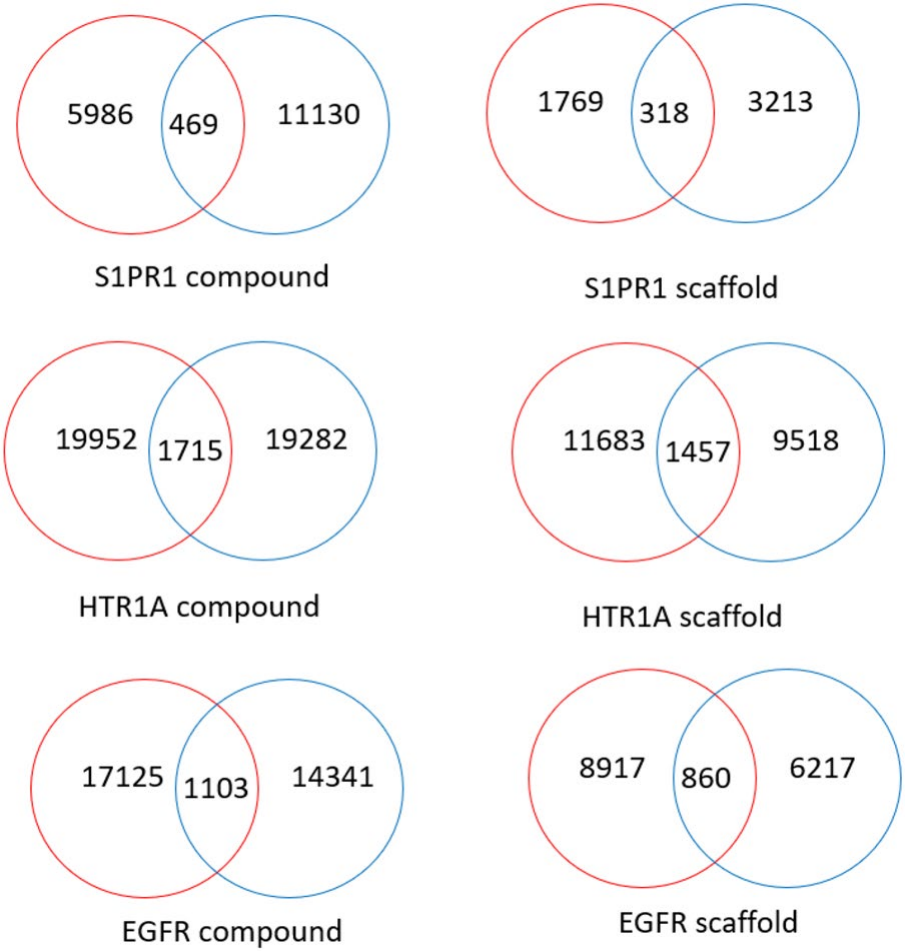
Venn diagram of LatentGAN (red) and RNN (blue) active compounds/scaffolds

Another interesting question to answer is if the LatentGAN can be trained to generate target specific compounds. The active compounds of training set were then used as the real data to train the LatentGAN. Each GAN model was trained 10,000 epochs and once the training was finished, 50,000 compounds were sampled from the generator and decoded with the heteroencoder. Then, three targets (EGFR, HTR1A and S1PR1) were selected and SVM target prediction models were built (see methods) to predict target activity on each target using the corresponding model (Table 3). Results show that in all cases validity was above 80% and the uniqueness of valid compound was 56%, 66% and 31% for EGFR, HTR1A and S1PR1 respectively. Comparing with the sample set of ChEMBL model these numbers are much lower, but this can be due to the smaller size of training sets. Additionally, RNN models with transfer learning trained on the three targets (see “Methods and materials”) show a higher percentage of validity, but their percentage of uniqueness is lower in all cases except for S1PR1. Regarding the novelty, the values are 97%, 95% and 98% for EGFR, HTR1A and S1PR1 respectively and are slightly higher than the values of the RNN transfer learning models. This demonstrates that LatentGAN not only can generate valid SMILES but also most of them are novel to the training set, which is very important for de novo design tasks. All the sampled valid SMILES were then evaluated by the SVM models and a high percentage of the LatentGAN generated ones were predicted as active for these three targets (71%, 71% and 44%, for EGFR, HTR1A and S1PR1 respectively). These scores were better than the RNN models with respect to EGFR, but worse with respect to other two. Additionally, the comparison between LatentGAN and RNN generated active structures (Fig. 3) shows that the overlap is very small between the two architectures at both compound and scaffold levels. The compounds generated by LatentGAN were evaluated using the RNN model for a probabilistic estimation of whether the RNN model eventually would cover the LatentGAN output space, and it was shown to be very unlikely (see Additional file 1). This highlights that both architectures can work complementarily.

**Table 3:** Metrics obtained from a 50,000 SMILES sample of all the models trained

Dataset	Arch.	Valid (%)	Unique (%)	Novel (%)	Active (%)	Recovered actives/total actives (%)	Recovered neighbors
EGFR	GAN	86	56	97	71	5.26	196
	RNN	96	46	95	65	7.74	238
HTR1A	GAN	86	66	95	71	5.05	284
	RNN	96	50	90	81	7.28	384
S1PR1	GAN	89	31	98	44	0.93	24
	RNN	97	35	97	65	3.72	43

Dataset used (Dataset), Architecture used (Arch.), Percent of valid molecules in the sampled set (Valid), Percent of valid unique compounds (Unique), Percent of unique novel (not present in the training set) compounds (Novel), Percent of unique active compounds (Active), Recovered actives from the test set given the entire number of actives in the test set (Recovered actives/Total Actives), Recovered neighbors of active compounds using FCFP6 fingerprint with 2048 bits and a threshold Tanimoto similarity of 0.7

**Fig. 4:**
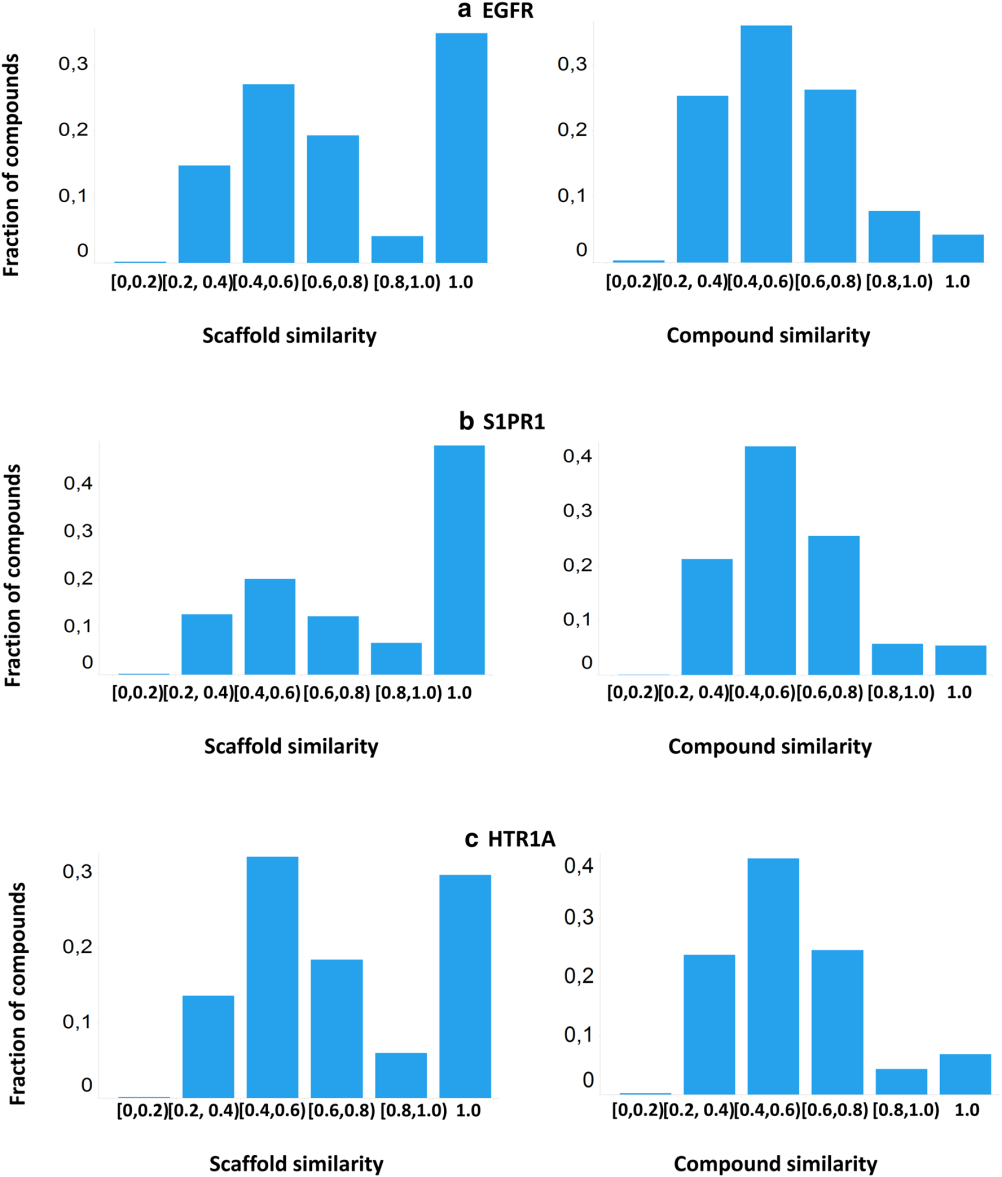
The distribution of Murcko scaffold similarity (left) and FCFP6 Tanimoto compound similarity (right) to the training set of molecules generated by LatentGAN models for **a** EGFR, **b** S1PR1 and **c** HTR1A

Full compound and Murcko scaffold [53] similarity was calculated between the actives in the sampled set and the actives in training set. Results (Fig. 4) show that, for each target, there are around 5% of generated compounds that are identical to the training sets. Additionally, there are around 25%, 24% and 21% compounds having similarity lower than 0.4 to the training set in EGFR, HTR1A and S1PR1 respectively. This means that LatentGAN is able to generate very dissimilar compounds to the training set. In terms of scaffold similarity comparison, it is not surprising that the percentage of scaffolds identical to the training set is much higher for all the targets. Nevertheless, around 14% of scaffolds in the sample set have low similarity to the training set (< 0.4) for all three tested cases.

**Fig. 5:**
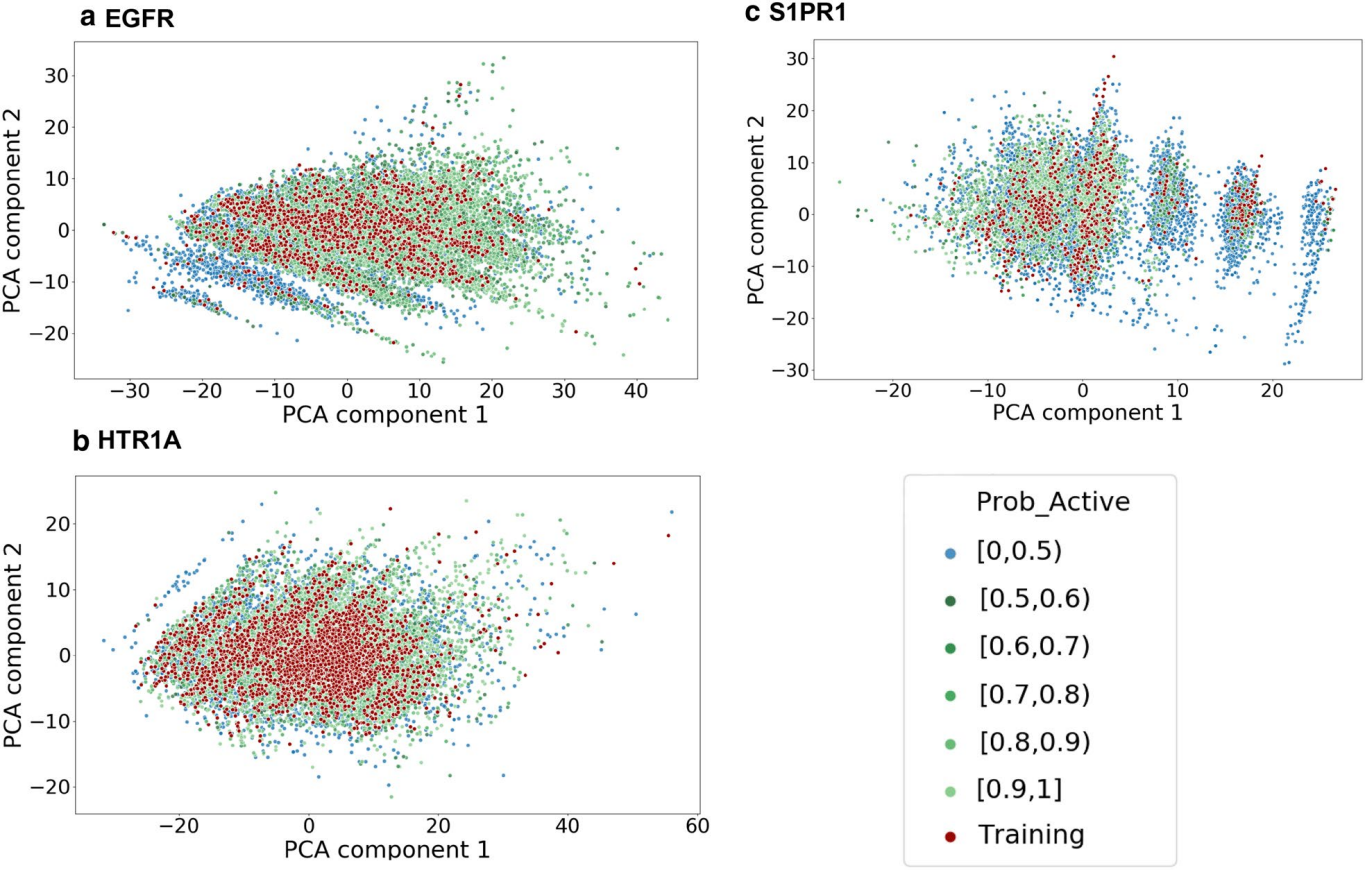
PCA analysis for **a** EGFR (explained variance 82.8%), **b** HTR1A (explained variance 75.0%) and **c** S1PR1 (explained variance 79.3%) dataset. The red dots are the training set, the blue dots are the predicted inactive compounds in the sampled set and other dots are the predicted actives in the sampled set with different level of probability of being active

**Fig. 6:**
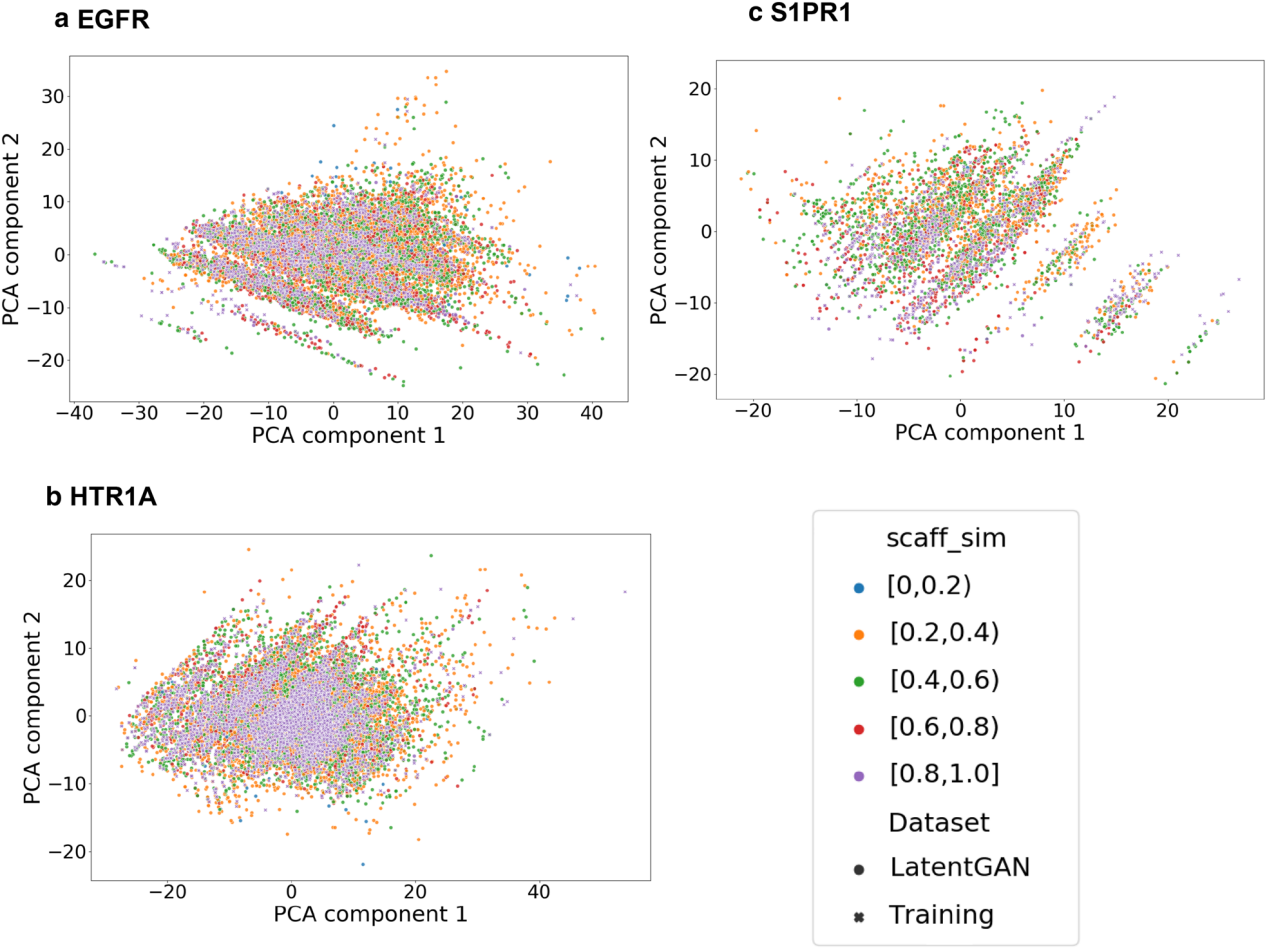
The same PCA analysis, showing the Murcko scaffold similarities of the predicted active compounds for **a** EGFR (explained variance 80.2%), **b** HTR1A (explained variance 74.1%) and **c** S1PR1 (explained variance 71.3%). Note that due to the lower amount in the outlier region of **c**, the image has been rotated slightly. No significant relationship between the scaffold similarities and the regions was found. For a separation of the generated points by similarity interval, see Additional file 1

**Fig. 7:**
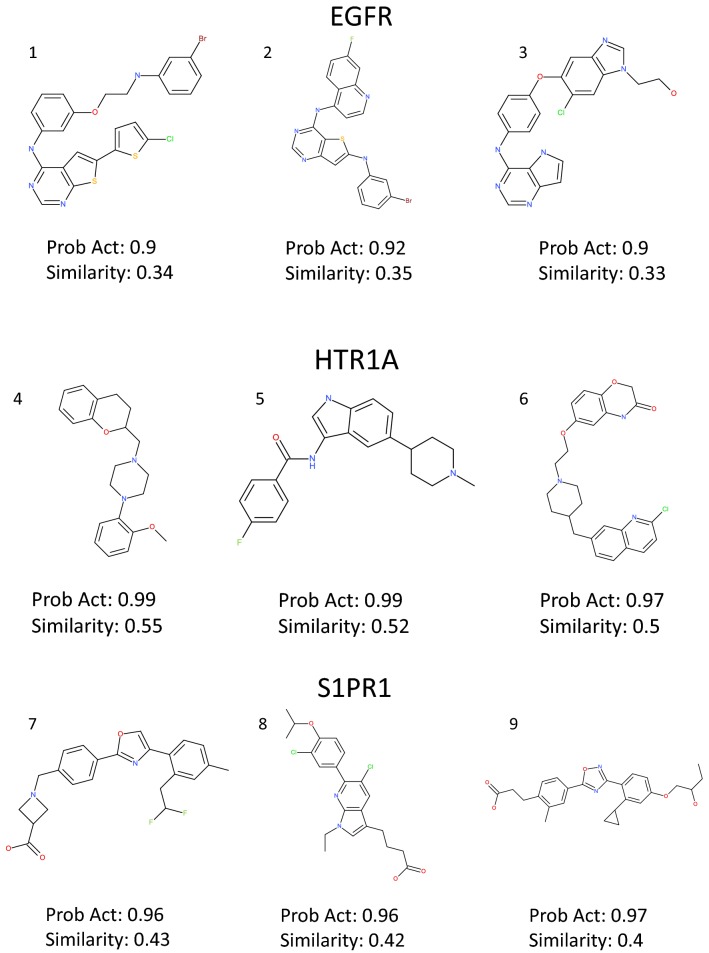
Examples generated by the LatentGAN. Compound 1-3 are generated by the EGFR model, 4–6 are generated by HTR1A model and 7–9 are generated by S1PR1 model

**Fig. 8:**
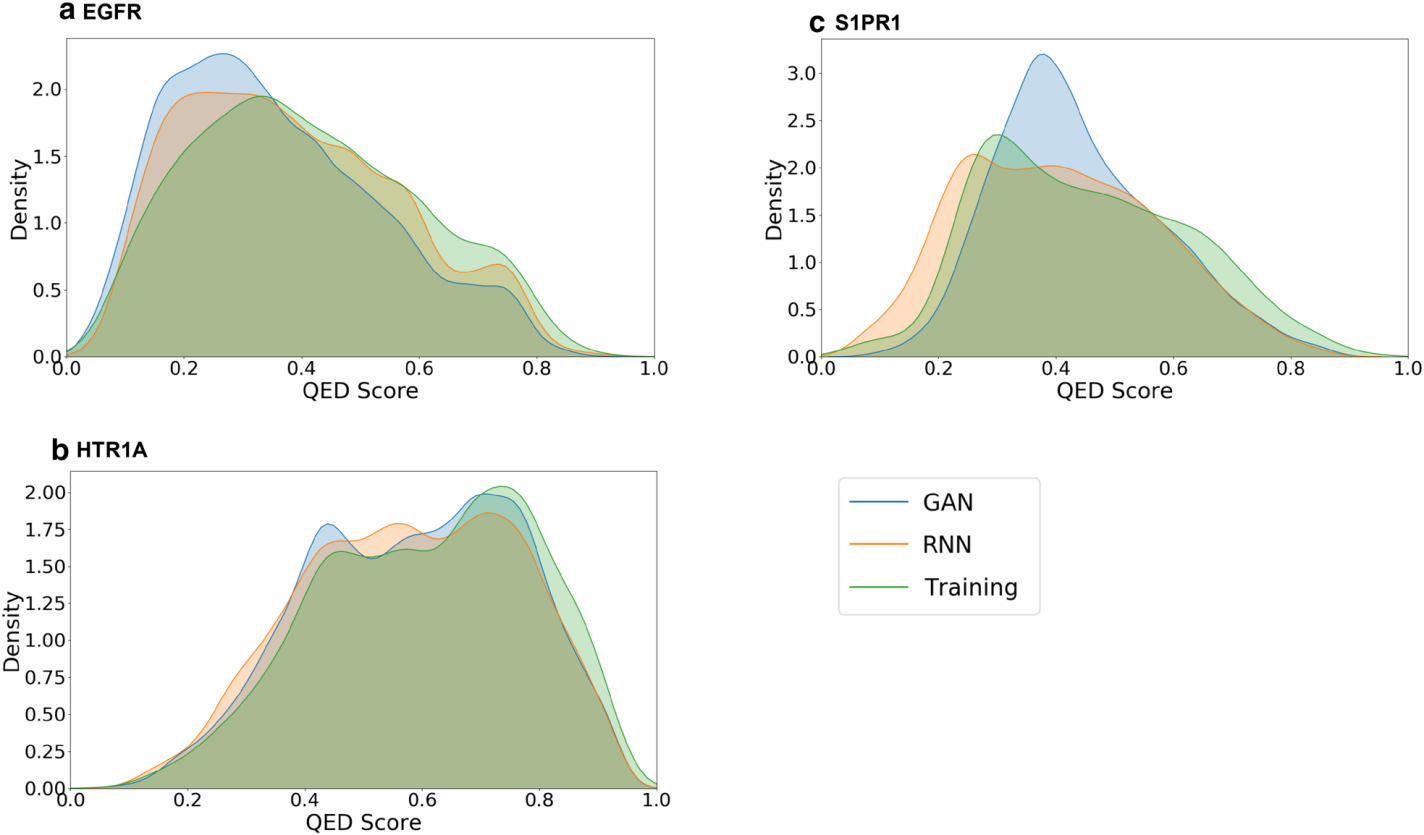
QED distributions of sampled molecules from EGFR (**a**), HTR1A (**b**) and S1PR1 (**c**)

**Fig. 9:**
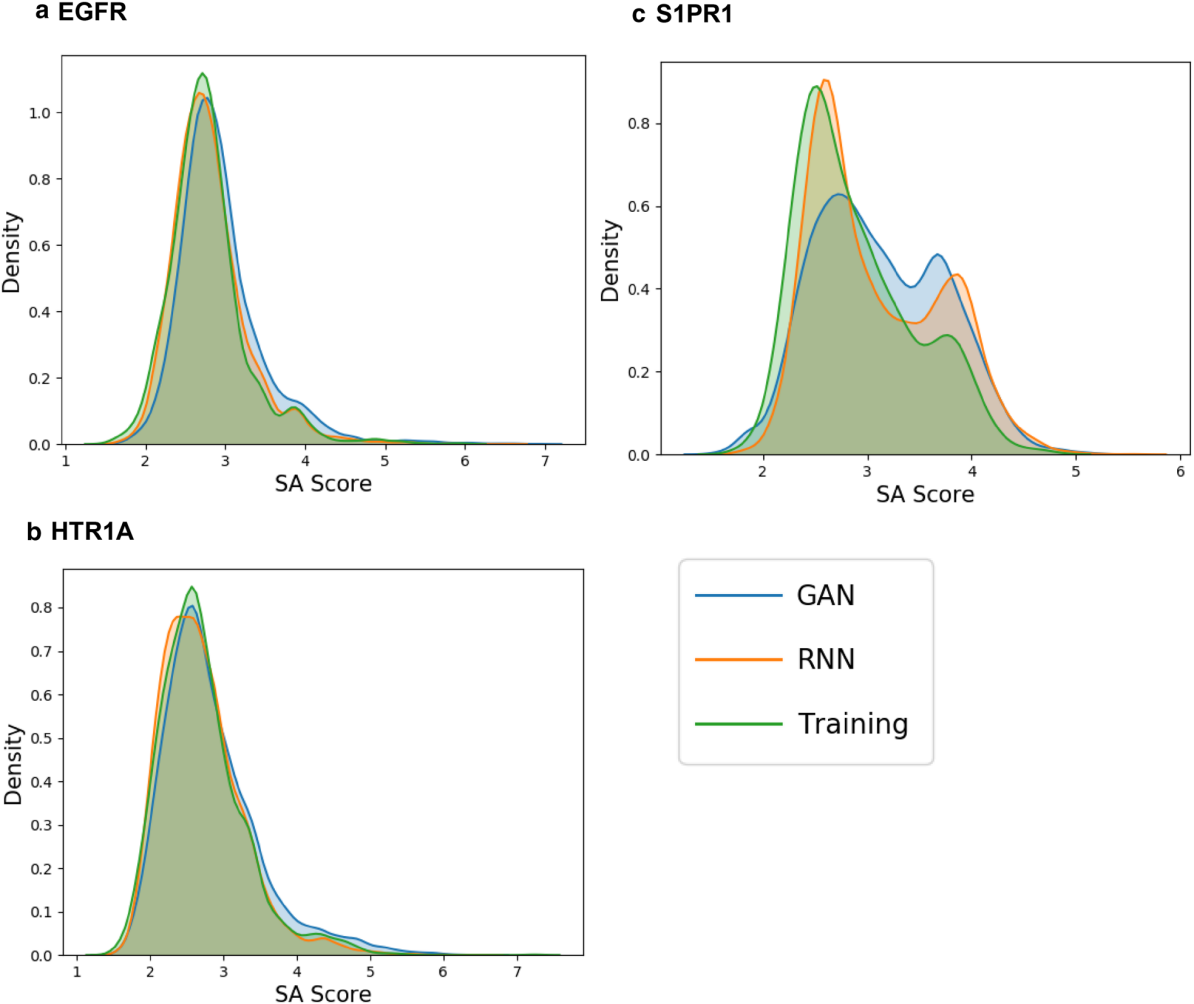
SA distributions of sampled molecules from EGFR (**a**), HTR1A (**b**) and S1PR1 (**c**)

A PCA analysis using the MQN fingerprint was performed to compare the chemical space of sampled sets and training sets of all targets and shows that the sampled compound sets cover most of the chemical space of the training sets (Fig. 5). Interestingly, there are some regions in the PCA plots where most of the sampled compounds around the training compounds are predicted as inactive, for example the left lower corner in EGFR (Fig. 5a) and the right-hand side region in S1PR1 (Fig. 5c). The training compounds in those regions are non-druglike compounds and outliers in the training set and the SVM models predicted them as inactive. No conclusive relationship between these regions of outliers and the scaffolds of lower similarity (Fig. 6). Additionally, we also evaluated the amount of the actives in the test set recovered by the sample set (Table 3). It is interesting to note that there are more active compounds belonging to the test set recovered by RNN model for all three targets, indicating that using multiple types of generative model for structure generation can be a viable strategy. Lastly, some examples generated by LatentGAN were drawn (Fig. 7) and the QED drug-likeness score [11] and Synthetic Accessibility (SA) score [54] distributions for each of the targets were plotted (Figs. 8 and 9, respectively). Training set compounds have a slightly higher drug-likeness, yet the overall distributions are similar, showing that LatentGAN models can generate drug-like compounds.

### Comparison with similar generative networks

The LatentGAN was assessed using the MOSES benchmark platform [48], where several generative metrics are used to evaluate the properties of molecular generative networks on a sample of 30,000 SMILES after training on a canonical SMILES subset of the ZINC database [55] of size 1,584,663. The full table of results for the MOSES benchmark is maintained and regularly updated at [56]. When compared to the similar structured networks of VAE, JTN-VAE [20] and AAE, it is noticeable that VAE model have an output distribution that has a significant overlap with the training set, as shown by the high scores of most test metrics (where the test set has a similar distribution to the training set) and the low novelty, indicating a mode collapse. When compared against the JTN-VAE and AAE models, the LatentGAN has shows comparable or better results in the Fréchet ChemNet Distance (FCD) [57], Fragment (Frag) and Scaffold (Scaf) similarities, while producing slightly worse results in the cosine similarity to the nearest neighbor in the test set (SNN).

### On the properties of autoencoder latent spaces

In earlier VAE or AAE based architectures for generative molecular models, the role of the encoder is to forcefully fit the latent space of the training data to a Gaussian prior [47] or at least some continuous distribution [9], achieved in the latter with a loss function based on Kullback–Leibler (KL) divergence [58]. This requires the assumption that by interpolating in the latent space between two molecules, the decoded molecule would then have either a structure or property that also lies between these molecules. This is not an intuitive representation, as the chemical space is clearly discontinuous—there is nothing between e.g. C_4_H_10_ and C_5_H_12_. The LatentGAN heteroencoder instead makes no assumption with regards to the latent space as no ground truth exists for this representation. Instead it is trained based strictly on the categorial cross entropy loss of the reconstruction. The result in a space of encoded latent vectors that the GAN later trains on that does not necessarily have to be continuous.

The complexity of the SMILES representation can also be a problem the training, as molecules of similar structures can have very different canonical SMILES when the starting atom changes, resulting in dissimilar latent representations of the same molecule. By training on non-canonical (random) SMILES [14, 21], this issue is alleviated since different non-canonical forms of the same molecule are encoded to the same latent space point which furthermore leads to a more chemically relevant latent space [22]. In addition, the multiple representations of the same molecule during training reduces the risk of overfitting the conditional probabilities of the decoder towards compounds who share a common substring of the SMILES in the canonical representation.

## Conclusions

A new molecule de novo design method, LatentGAN, was proposed by combining a heteroencoder and a generative adversarial network. In our method, the pretrained autoencoder was used to map the molecular structure to latent vector and the GAN was trained using latent vectors as input as well as output, all in separate steps. Once the training of the GAN was finished, the sampled latent vectors were mapped back to structures by the decoder of the autoencoder neural network. As a first experiment, after training on a subset of ChEMBL compounds, the LatentGAN was able to generate similar drug-like compounds. We later applied the method on three target biased datasets (EGFR, HTR1A and S1PR1) to investigate the capability of the LatentGAN to generate biased compounds. Encouragingly, our results show that most of the sampled compounds from the trained model are predicted to be active to the target which it was trained against, with a substantial portion of the sampled compounds being novel with respect to the training set. Additionally, after comparing the structures generated from the LatentGAN and the RNN based models for the corresponding targets, it seems that there is very little overlap among the two sets implying that the two types of models can be complementary to each other. In summary, these results show that LatentGAN can be a valuable tool for de novo drug design.

## Supplementary information

**Additional file 1.** Supplementary figures and table.

## Data Availability

The training sets and the trained heteroencoder model version used is available in through a GitHub repository (https://github.com/Dierme/latent-gan), which also contains the source code of the LatentGAN.
