# Supplementary material for: A de novo molecular generation method using latent vector based generative adversarial network
Source: J Cheminform. 2019 Dec 3;11:74. doi: 10.1186/s13321-019-0397-9 (PMC6892210; doi:10.1186/s13321-019-0397-9)
Supplement: Supplementary file 1 — Additional file 1. Supplementary figures and table. [file 13321_2019_397_MOESM1_ESM.docx]

**Additional material**

Oleksii Prykhodko^€,¥,∆^, Simon Johansson^€,¥,∆,*^, Panagiotis-Christos Kotsias^∆^, Josep Arús-Pous^∆§^, Esben Jannik Bjerrum^∆^, Ola Engkvist^∆^, Hongming Chen^∆,*,†^

∆ Hit Discovery, Discovery Sciences, Biopharmaceutical R&D, AstraZeneca, Gothenburg, Sweden

§ Department of Chemistry and Biochemistry, University of Bern, Switzerland

¥ Department of Computer Science and Engineering, Chalmers University of Technology, Gothenburg, Sweden

† Chemistry and Chemical Biology Centre, Guangzhou Regenerative Medicine and Health-Guangdong Laboratory, Science Park, Guangzhou, China

€ Authors contribute equally

* Corresponding authors:

[Simon.johansson@astrazeneca.com](mailto:Simon.johansson@astrazeneca.com)

[Hongming.chen71@hotmail.com](mailto:Hongming.chen71@hotmail.com)

**Figures of PCA plots for Murcko Scaffold Similarity**

**Figure S1:** PCA plot of the predicted active compounds for EGFR split by the interval of Murcko scaffold similarity.

**Figure S2:** PCA plot of the predicted active compounds for HTR1A split by the interval of Murcko scaffold similarity.

**Figure S3:** PCA plot of the predicted active compounds for S1PR1 split by the interval of Murcko scaffold similarity.

**A probabilistic assessment of the overlap between RNN and LatentGAN.** Using the RNN model, we computed the negative log likelihood (NLL) of generating each SMILES string from both the RNN and the LatentGAN output sample and compared the means NLL for the three targets (EGFR, HTR1A, S1PR1). These are shown in Table S1 below.

**Table S1**. Mean NLLs of generated sets using the RNN model with the corresponding transfer learning.

| Target | RNN | LatentGAN |
| --- | --- | --- |
| EGFR | 16.1 | 40.1 |
| HTR1A | 15.2 | 32.8 |
| S1PR1 | 16.3 | 35.8 |

This means, that for e.g. HTR1A, where the difference in mean NLL is 17.6, that on average a molecule generated by the LatentGAN is $e^{17.6}\approx44\cdot{10}^{6}$ times less likely to be generated by the RNN than the average molecule generated by the RNN. We make no claims regarding the size of the output space and therefore do not comment the actual frequency of the generated compounds.
